# Cerebral vasculitis mimicking intracranial metastatic progression of lung cancer during PD-1 blockade

**DOI:** 10.1186/s40425-017-0249-y

**Published:** 2017-06-20

**Authors:** Heinz Läubli, Jürgen Hench, Michal Stanczak, Ingmar Heijnen, Alexandros Papachristofilou, Stephan Frank, Alfred Zippelius, Frank Stenner-Liewen

**Affiliations:** 1 Department of Internal Medicine, Division of Medical Oncology, University Hospital Basel, Basel, Switzerland; 2 Department of Biomedicine, Cancer Immunology Laboratory, University Hospital Basel, Basel, Switzerland; 3 Department of Pathology, Division of Neuropathology, University Hospital Basel, Basel, Switzerland; 4 Department of Laboratory Medicine, University Hospital Basel, Basel, Switzerland; 5 Department of Radiology, Division of Radiotherapy, University Hospital Basel, Basel, Switzerland; 6 Medical Oncology and Cancer Immunology, University Hospital Basel, Petersgraben 4, 4031 Basel, Switzerland

**Keywords:** Checkpoint inhibitor, Cancer immunotherapy, Autoimmune, Antibody, Brain metastasis

## Abstract

**Background:**

Stimulation of the immune system by targeting the PD-1/PD-L1 pathway can result in activation of anti-tumor immunity. Besides its clinical benefit immune checkpoint therapy leads to significant immune-related adverse events (irAEs). Some rare irAEs are not well described yet but are critical in patient management.

**Case presentation:**

Here, we describe a case of autoimmune cerebral vasculitis/encephalitis after PD-1 inhibitor treatment for metastatic adenocarcinoma of the lung. Upon PD-1 blockade, the patient developed cerebral lesions, while having disease stabilization of extracranial metastases. Imaging suggested that the patient had new progressing brain metastases. Despite stereotactic irradiation the lesions progressed further. The largest lesion became symptomatic and had to be surgically resected. On examination, cerebral vasculitis was detected but not evidence of metastatic lung cancer. Analysis of the patient’s serum revealed the presence of antinuclear antibodies that were already present before starting PD-1 blockade. In addition, we also found anti-vascular endothelial antibodies in the serum.

**Conclusion:**

This finding suggests that the patient had preformed autoantibodies and the checkpoint inhibitor induced a clinically relevant autoimmune disease. Taken together, encephalitic lesions in patients under PD-1/PD-L1 blockade can mimic metastatic brain lesions and this rare irAE has to be considered as a differential diagnosis in patients treated with immunotherapy.

## Background

Activation of the immune system against tumors with blocking antibodies that target immune-modulatory receptors on T cells have been successfully introduced into clinical oncological routine [1]. In particular, targeting of the programmed death-1 (PD-1) and the programmed death-ligand-1 (PD-L1) has shown considerable anti-tumor activity across multiple cancer entities including non-small cell lung cancer [1–3]. Awareness for the more common immune-related adverse events (irAEs) is growing, but uncommon events including immune-related affections of the central nervous system are still underestimated. These are difficult to diagnose and intracranial cancer progression is often assumed to be responsible for new cerebral lesions on magnetic resonance imaging (MRI).

## Case presentation

**Fig. 1:**
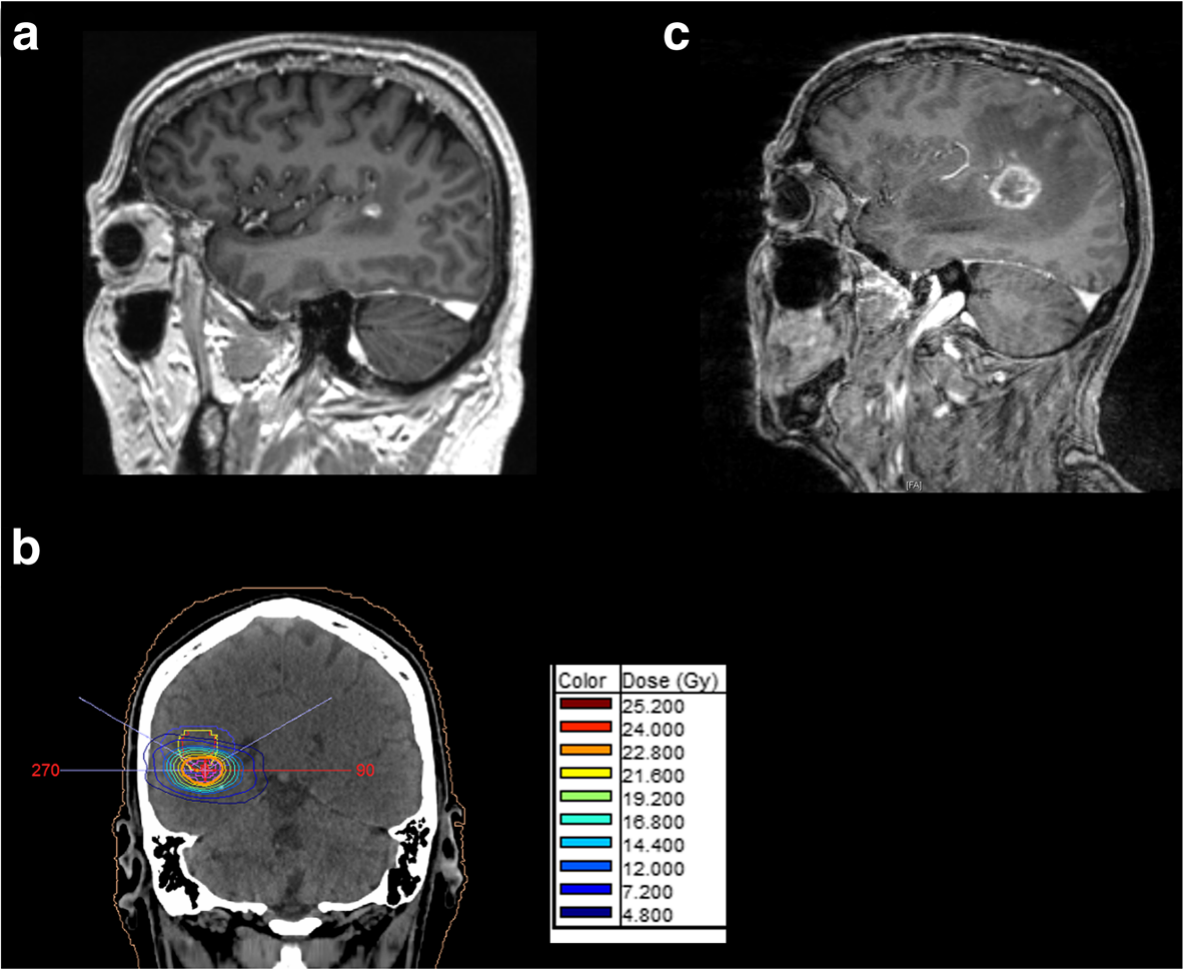
Magnetic resonance imaging of intracranial lesions. **a** Parieto-temporal lesion that was irradiated. T1 weighted MRI after application of Gadolinium-containing contras material. **b** Presentation of the radiation field that was applied to the lesion. **c** Progression of irradiated lesion after irradiation and before resection of the lesion

Here, we report on a 53-year old man with stage IV adenocarcinoma of the lung with no predictive genetic abnormalities (no EGFR mutation, no ALK or ROS1 translocation). On his initial computed tomography (CT) scan, he presented with multiple bilateral pulmonary nodules, masses in mediastinal lymph nodes, liver, and ribs. The patient had no history of auto-immune disorders. MRI of the skull revealed 2 cerebral lesions, which were treated by irradiation with one fraction of 20 Gray and showed consecutive regression. A subsequent palliative chemotherapy with cisplatin and pemetrexed led to disease stabilization after four cycles. Pemetrexed was continued as maintenance therapy. Two months later, progression of several lesions prompted a second line therapy with the PD-1 inhibitor nivolumab. Serological testing for human immunodeficiency, hepatitis B and C virus infections were negative. Under PD-1 blockade peripheral lesions regressed and quality of life improved. Soon thereafter, however, walking ability deteriorated, and cranial MRI showed a new parieto-temporal lesion in proximity of the formerly irradiated masses (Fig. 1a). Suggestive of further metastatic spread, this new lesion was irradiated with 24 Gray over 7 days (Fig. 1b), and nivolumab continued. A few days later, the patient was admitted to the emergency department with progressive gait disturbance and speech difficulties. He had no clincial symptoms for auto-immune disorders such as a systemic lupus erythematodes or generalized vasculitis. Corticosteroid treatment was initiated and provided partial relief only. MRI demonstrated progression of the newly irradiated lesion (Fig. 1c), whereas extracerebral masses remained stable. The progressive brain lesion was surgically removed and subjected to neuropathological examination.

**Fig. 2:**
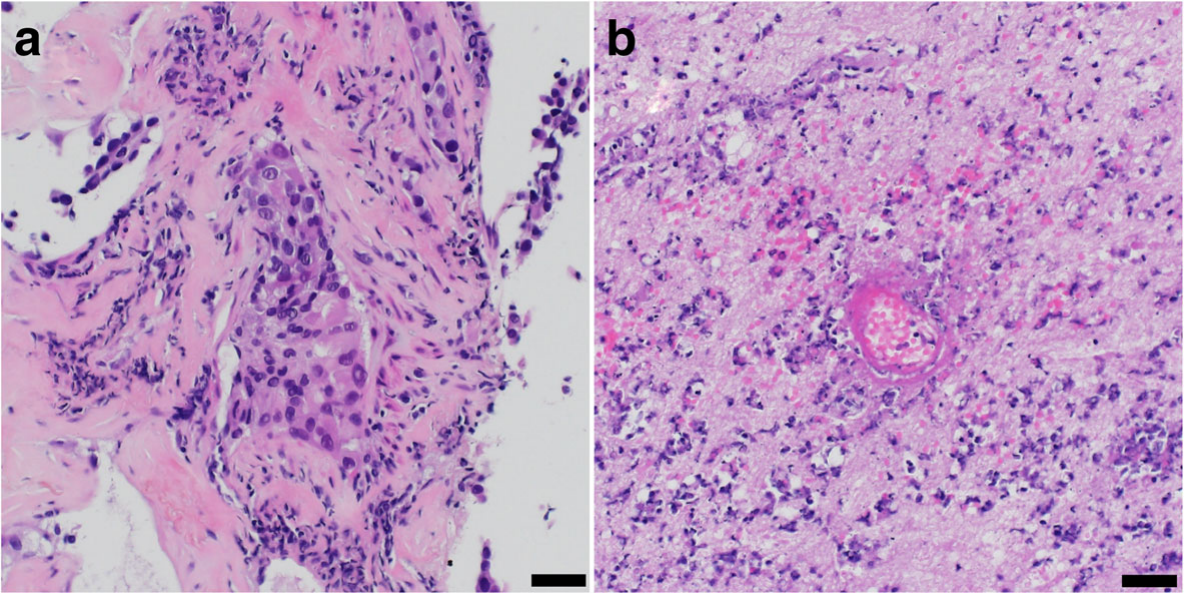
Histological analysis of the resected lesion. H&E stained specimens. **a** Initial biopsy from bronchial lesion. **b** Resected cerebral lesion reveals necrotic brain tissue with vasculitic inflammatory infiltrates. Scale bars: 20 μm

**Fig. 3:**
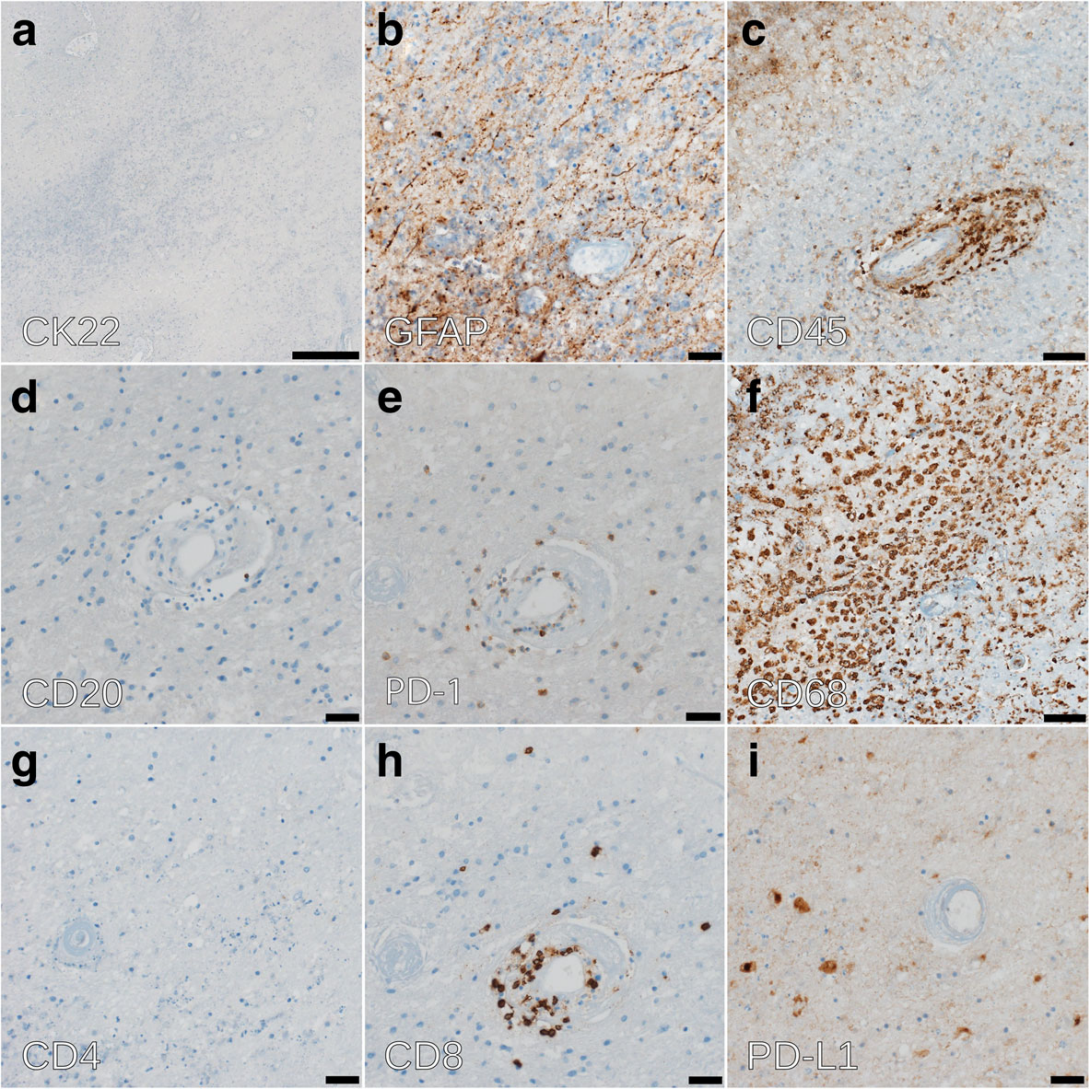
Immunohistochemical analysis of brain lesion. **a** Staining with CK22 monoclonal anti-cytokeratin antibody cocktail. **b** Staining with the astroglial marker anti-GFAP (glial fibrillary acidic protein). Scale bar: 20 μm. **c** CD45 (leukocyte antigen) staining for the detection of immune cells demonstrates perivascular infiltrates. Scale bar: 50 μm. **d** Very few CD20-positive B cells are detected. Scale bar: 20 μm. **e** The perivascular infiltrate are predominantly PD-1 positive T cells. Scale bar: 20 μm. **f** CD68 staining of macrophages highlights resorptive changes. Scale bar: 50 μm. (**g**, **h**) The perivascular T cell infiltrates are mainly CD8 T cells. Scale bar: 20 μm. **i** Only little PD-L1 staining was detected. Scale bar: 20 μm

Histopathological analysis revealed necrotizing encephalitis with no evidence of metastatic lung cancer (Fig.2a and b). The dimensions correlated with the radiographic size of the lesion (diameter approximately 3.3 cm). Staining for cytokeratins (CK22), performed on all paraffin embedded specimens was negative (Fig. 3a). A high perivascular density of CD45 positive cells was found (Fig. 3c) with predominance of PD-1 positive T cells over B cells (CD20, PD-1, CD4 and CD8 staining, Fig.3d, e, g and h). Staining of CD68 demonstrated accompanying resorptive changes of the brain tissue (Fig. 3f). We also found a predominance of CD8 positive T cells over CD4 positive T cells (Fig. 3g and h). PD-L1 expressing cells were sparse (Fig. 3i). Further analysis of infectious diseases including Toxoplasma were negative (not shown). Parallel panel sequencing (Oncomine™ Comprehensive Cancer Panel, Thermo Fisher) was performed on the initial lung biopsy as well as on DNA extracted from the necrotizing encephalitis. Most prevalent mutations identified in the primary tumor and present in ATM, TP53, and NOTCH1 (Table 1) could not be detected in the brain lesion. This result confirms our histological findings, making it unlikely that tumor cells had been present in the biopsied brain region. We hypothesized that the brain lesion was the consequences of an autoimmune phenomenon during PD-1 blockade.

**Table 1:** Results of NGS analysis from primary tumor of the patient

Genes	Amino Acid Change	Allele Ratio
ATM^a^	p.Asp1853Asn	G = 0.57, A = 0.43
BRCA1	p.Asn1052Asp	T = 0.8421, C = 0.1579
BAP1	p.Arg114Cys	G = 0.8783, A = 0.1217
TSC1	p.Lys106Arg	T = 0.8925, C = 0.1075
TP53^a^	p.Ala159Pro	CGCGGACGCGGGT = 0.7869, GGCGGACGCGGGT = 0.2131
MIR4673,NOTCH1^a^	p.Gly347Asp	C = 0.7327, T = 0.2673
CDH1	p.Val556Leu	G = 0.9169, T = 0.0831
TP53	p.Gly266Ter	C = 0.9294, A = 0.0706, G = 0.0, T = 0.0
CDKN2A	p.Ser7fs	TC = 0.7684, T = 0.2, TT = 0.0316
MSH2	p.Ser734Phe	C = 0.9257, T = 0.0743
PTCH1	p.Gly1299Asp	C = 0.9205, T = 0.0795

^a^tumor-associated mutations with highest frequency

**Fig. 4:**
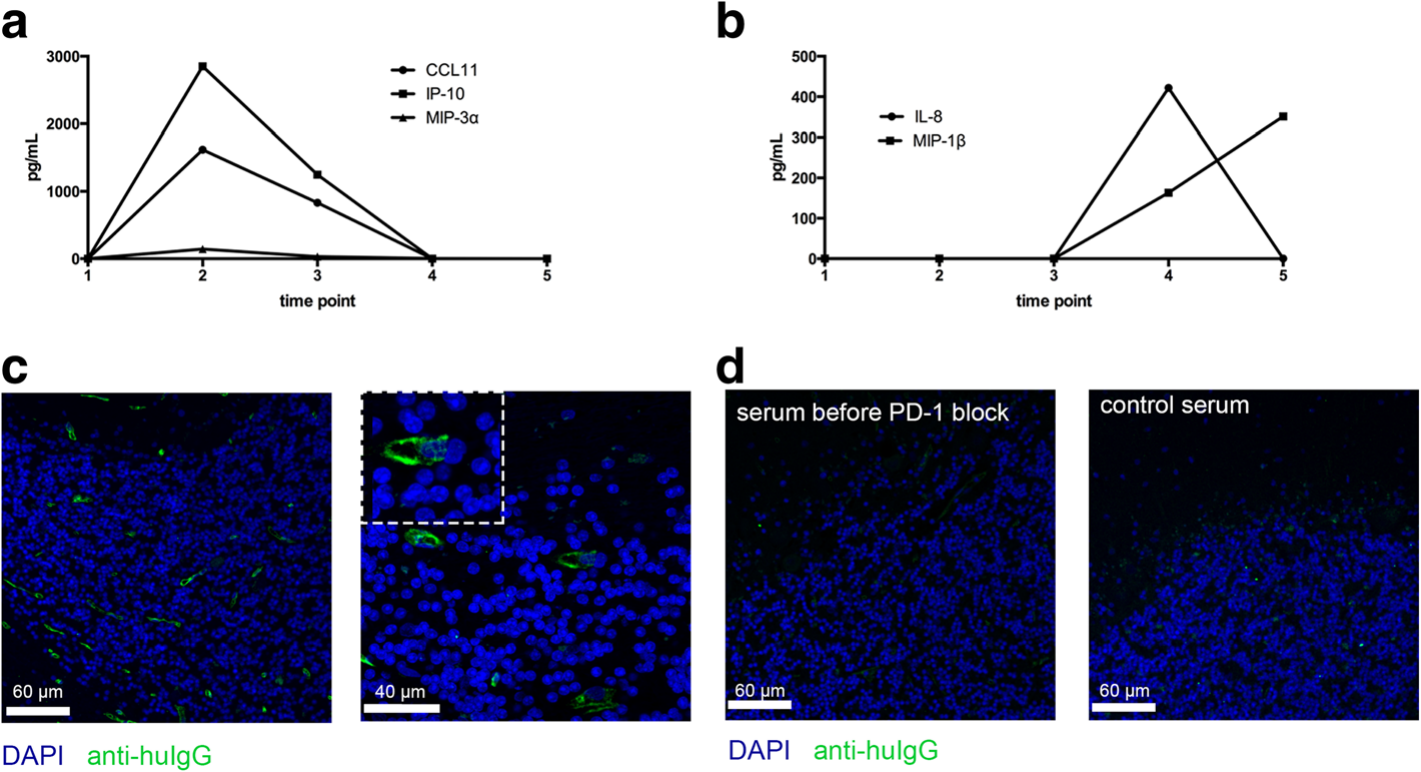
Chemokine levels over time and detection of anti-vascular endothelial antibodies. **a**, **b** Changes in inflammatory chemokine levels that were measured in the serum of the patient by a multiplex bead assay. Chemokines were measured before treatment with nivolumab (time point 1), after 4 weeks (time point 2), after 10 weeks (time point 3), at the diagnosis of the vasculitis/encephalitis (time point 4), and 3 weeks later (time point 5). **c** Staining of control cerebellum sections with serum from the patient at the time point when the vasculitis/encephalitis was diagnosed. **d** Staining of cerebellar sections with control serum (*left panel*) or serum from the patient before PD-1 blockade (*right panel*)

To evaluate for a potential autoimmune syndrome, we screened for anti-neuronal autoantibodies, as well as antinuclear antibodies (ANA). No antibodies against neuronal antigens (i.e., Hu, Yo, Ri, CV2, Ma1, Ma2/Ta, and amphiphysin, NMDA, GAD65) could be detected, but high titers of antinuclear anti-SSA/Ro and anti-SSB/La antibodies were found (>240 U/mL, reference <10 U/mL). High antibody titers against SSA/Ro (Ro52, Ro60) were already found in serum samples that were taken before the initiation of anti-PD-1 treatment. Inflammatory chemokines and cytokines in serum were measured by a flow cytometry bead assay that allows measurement of 13 inflammatory chemokines or cytokines simultaneously (both from Biolegend). Binding of chemokines/cytokines to the beads were measured by a Fortessa analyzer (BD Biosciences). Multiplex analysis of serum samples showed a measurable level of TNFα at the time point when the encephalitis was diagnosed (25.4 pg/mL). At other time points, the TNFα level was below the detection limit. Measurement of inflammatory chemokines showed an increase of blood levels upon treatment initiation with anti-PD-1 antibody including CCL11, IP-10, and MIP-3α (Fig. 4a). Only MIP-1β and IL-8 were increased at the time point of resection of the encephalitic lesion (Fig. 4b). The increase in inflammatory chemokines and also TNFα in our patient supported the presence of an inflammatory process. Moreover, when histological cerebellum sections from an unrelated healthy control individual were incubated with patient’s serum taken at the time point when the vasculitis was diagnosed, a strong staining of the endothelial layers of cerebellar vessels was found (Fig. 4c). Testing of control cerebellar sections with control serum or patient’s serum before nivolumab treatment showed no staining (Fig. 4d). However, increasing concentrations with pre-PD-1 serum from the patient led to some but overall weaker endothelial staining (not shown) indicating that the anti-vascular antibodies were present before PD-1 blockade, but the titer was lower.

The patient’s neurological symptoms rapidly improved after excision of the lesion. Nivolumab treatment was stopped and corticosteroids were tapered over the course of two months. Follow-up imaging of head and chest showed slowly progressive pulmonary lesions and a new neocortical contrast agent-enhancing lesion, which was asymptomatic at the time of imaging. This new lesion was interpreted as encephalitis, and corticosteroids were administered again. In parallel, a second line chemotherapy with docetaxel was initiated. After three cycles of chemotherapy, restaging showed a partial response of the lung lesions and a regression of the cerebral lesion.

## Discussion

This case demonstrates that rare side effects of PD-1 blockade have to be considered when treating patients with this novel immunotherapeutic. Neurological side effects of immune checkpoint blockade have rarely been reported in large randomized phase III trials [2–7]. However, several cases of central nervous system disorders have been described in the literature [8–10]. In one case, an autoimmune limbic encephalitis was diagnosed in a patient with metastatic melanoma treated with the PD-1 blocker pembrolizumab for 1 year [9]. Treatment of this patient with corticosteroids could prevent the progression of the encephalitis but left the patient with residual neurological symptoms. In another case, a small vessel vasculitis was diagnosed in melanoma patient undergoing treatment with pembrolizumab [8]. The patient presented with eosinophilic fasciitis after being treated for 18 months with PD-1 blockade. The patient developed neurological symptoms including confusion and gait disturbances [8]. In another case, an encephalitic lesion developed under PD-1 blockade with pembrolizumab (then still termed lambrolizumab) [10]. The patient was only treated with anti-epileptics for his seizures and the symptoms resolved after stopping the checkpoint inhibitor.

Our case highlights the difficulty in differentiating between cancer progression and irAE, in particular if the irAE is rare and the likelihood of cancer progression is high. Encephalitis due to PD-1/PD-L1 blockade is a very uncommon but severe complication. The description of such rare cases is important to establish the awareness for the differential diagnosis. In addition, pseuprogression needs to be consideres although the clinical course and the lack of CK22 positive cells in histology makes this diagnosis unlikely in this case. Morevoer, the absence of tumor-associated mutations by sensitive NGS analysis suggests that the lesion developed due to an auto-immune cerebral vasculitis triggered by PD-1 blockade. Concomitant irradiation might also have triggered the development of autoimmune encephalitis. Combination of radiotherapy with PD-1 blockade is supposed to act synergistically, in part due to immunogenic cell death and enhanced uptake of antigens from irradiated tumor cells by antigen presenting cells [11–13]. This finding has been investigated in preclinical models [13, 14], and observational data also suggests clinical efficacy in cancer patients [11, 12]. In order to better differentiate between inflammatory lesions and cancer progression in patients undergoing checkpoint blockade, better diagnostic tools should be developed. While fluorodesoxy-glucose positron emission tomography cannot segregate between inflammatory and cancer lesions, the quantification of tumor-derived DNA in serum or in our case the cerebro-spinal fluid could help to find the right differential diagnosis.

We found anti-vascular IgG antibodies in the serum of our patient when the diagnosis of cerebral vasculitis/encephalitis was made. Analysis of human brain endothelial cells has shown an expression of PD-L2 [15]. The investigators also demonstrated that PD-L2 inhibits transmigration of CD4 and CD8 T cells [15]. PD-1 blockade by antibodies such as pembrolizumab or nivolumab affects interactions with PD-L1 and PD-L2, whereas PD-L1 blockade with antibodies such as atezolizumab or durvalumab is not targeting PD-L2-mediated interactions. Thus, patients under PD-1 blockade might be more prone to irAE that involve intracerebral blood vessels compared to PD-L1 blockade. Also in support of a function of PD-1-mediated interactions in neuroinflammation is the finding that polymorphisms of PD-1 have been linked to multiple sclerosis [16]. In our case antinuclear anti-SSA/Ro antibodies were present before PD-1 blockade. At this time it remains unclear if patients with ANA are at higher risk to develop irAEs. We can also not exclude that the propensity of this patient to an auto-immune reaction was due to a paraneoplastic syndrome, since we had no blood sample for analysis available before the patient had cancer. Moreover, it would be interesting to perform an analysis on sera from a cohort of patients during PD-1/PD-L1 blockade and correlate the presence of ANA with irAEs. In general, an increased risk to develop cancer was reported in patients with anti-SSA/Ro antibodies [17]. Patients aged 55 years or older and with cutaneous lupus erythematodes that had anti-SSA/Ro antibodies had a higher risk to develop malignancies including bronchial carcinoma [17]. Patients undergoing PD-1/PD-L1 directed therapy with pre-existing autoimmune disease seem to develop more often irAEs. A recent analysis in Australia showed that 38% of 119 melanoma patients with pre-existing autoimmune disorder or with previous irAEs during ipilimumab therapy developed a flare that necessitated immunosuppression during PD-1 blockade [18]. However, autoimmunity was usually well manageable and only 2% had to definitively discontinue the treatment [18].

## Conclusion

In summary, we report a case of cerebral vasculitis accompanied by the development of anti-endothelial antibodies as a severe complication of PD-1/PD-L1-directed immunotherapy for lung cancer. Importantly, the necrotic brain lesion mimicked cancer progression with newly occurring intracerebral lesions. The presence of ANA prior to the start of PD-1 immunotherapy and the subsequent development of a severe irAEs warrants further investigation.

## Abbreviations

ANA: Antinuclear antibodies
irAE: Immune-related Adverse event
MRI: Magnetic resonance imaging
PD-1: Programmed Death-1
PD-L1 or –L2: Programmed Death Ligand-1 or -2
TNFα: Tumor Necrosis Factor α
